# Associations between hypertension and cognitive, mood, and behavioral parameters in very old adults: results from the IlSIRENTE study

**DOI:** 10.3389/fpubh.2023.1268983

**Published:** 2024-03-12

**Authors:** Helio José Coelho-Junior, Riccardo Calvani, Matteo Tosato, Andrea Russo, Francesco Landi, Anna Picca, Emanuele Marzetti

**Affiliations:** ^1^Department of Geriatrics, Orthopedics and Rheumatology, Università Cattolica del Sacro Cuore, Rome, Italy; ^2^Fondazione Policlinico Universitario “A. Gemelli” IRCCS, Rome, Italy; ^3^Department of Medicine and Surgery, LUM University, Casamassima, Italy

**Keywords:** cardiovascular disease, antihypertensive drugs, cognitive function, psychiatric disorders, depression, mood, aged

## Abstract

**Introduction:**

Studies on the associations between hypertension-related parameters and cognitive function, mood, and behavioral symptoms in older adults have produced mixed findings. A possible explanation for these divergent results is that investigations have not adequately adjusted their analyses according to the use of angiotensin-converting enzyme inhibitors (ACEIs). Therefore, the present study examined the cross-sectional associations between hypertension-related parameters, ACEI use, and cognitive function, mood, and behavioral symptoms in very old adults.

**Methods:**

This study was conducted by analyzing the IlSIRENTE database, a prospective cohort study that collected data on all individuals aged 80 years and older residing in the Sirente geographic area (*n* = 364). Blood pressure (BP) was assessed after 20 to 40 min of rest, while participants sat in an upright position. Drugs were coded according to the Anatomical Therapeutic and Chemical codes. Cognitive function, mood, and behavioral symptoms were recorded using the Minimum Data Set Home Care instrument. Blood inflammatory markers were measured.

**Results:**

Hypertension-related parameters were significantly associated with many cognitive, mood, and behavioral parameters after adjustment for covariates. However, only the inverse association between hypertension and lesser problems with short-term memory remained significant. Participants with hypertension had lower blood concentrations of inflammatory markers in comparison to their normotensive peers.

**Conclusion:**

Findings from the present study indicate that high BP values are associated with fewer complaints about memory problems in very old adults. Furthermore, a lower concentration of inflammatory markers was found in hypertensive participants. ACEI use might affect this scenario.

## 1 Introduction

Hypertension is a chronic condition characterized by sustained elevations in blood pressure (BP) (1). This disease is highly prevalent in older adults, affecting more than 70% of those aged 65 and over (2). These data deserve concern, given that uncontrolled hypertension predisposes to the occurrence of numerous undesirable health events, including stroke, myocardial infarction, and heart failure (1).

Besides the well-established associations between high BP levels and cardiovascular risk, considerable interest has been given to the possible effects of hypertension on brain functioning. As a matter of fact, many studies have found that the progression of hypertension is associated with cognitive decline and the occurrence of mild cognitive impairment (MCI) and dementia, including Alzheimer’s disease (AD) (3–5). However, these results are conflicting, given that other investigations found that high BP levels might be a protective mechanism, mainly in very old adults, to counteract the deleterious effects of aging on cerebrovascular structure (6–9).

The disturbance of the renin-angiotensin system is one of the major causes of hypertension (10). Augmented and uncontrolled production of angiotensin II (ANGII) is associated with detrimental effects in many organs, including heart, vessels, and kidneys (10, 11). In the brain, ANG II causes important structural and functional changes (12–14), which are exacerbated with aging (13). Treatment with angiotensin-converting enzyme inhibitors (ACEIs) might reduce the deleterious effects of ANGII on the brain in preclinical models (15–17). Such effect might occur through reductions in the inflammatory milieu (15–17).

To expand the knowledge on the subject, the present study was conducted to examine the associations between hypertension-related parameters and cognitive function, mood, and behavioral aspects in a well-characterized cohort of very old adults. The possible role of ACEIs in this relationship was also analyzed.

## 2 Methods

Data for the present investigation were obtained from the IlSIRENTE study database (18). IlSIRENTE was a prospective cohort study conducted in the mountain community of the Sirente geographic area (L’Aquila, Abruzzo) in Central Italy. The study was compliant with the principles of the Declaration of Helsinki and the protocol was approved by the Ethics Committee of the Università Cattolica del Sacro Cuore (Rome, Italy). All participants signed an informed consent prior to enrolment.

### 2.1 Study population

A list of all persons living in the Sirente area was obtained in October 2003 from the registry offices of the 13 municipalities involved in the study. Potential study participants were subsequently identified by selecting all those born before 1 January 1924 and currently living in that region. The total sample enrolled in the ilSIRENTE study consisted of 364 older adults.

### 2.2 Data collection

Baseline assessments began in December 2003 and were completed in September 2004. Follow-up visits took place after 24 months of baseline assessment. Information about medical history, medications, and lifestyle habits (e.g., smoking, alcohol consumption, physical activity) was collected using validated questionnaires (18). Body height and weight were measured through a stadiometer and an analog medical scale, respectively. The body mass index (BMI) was then calculated as the ratio between body weight (kg) and the square of height (m^2^).

### 2.3 Hypertension parameters

BP was measured after 20 to 40 min of rest using an aneroid sphygmomanometer and a stethoscope, while participants sat in an upright position. Three measurements were taken from the left arm, and only one from the right arm. BP values were then calculated from the average of the last two measures of the left arm plus the measure obtained from the right arm. Four hypertension groups were generated according to: (a) self-report of physician diagnosis (CLI-HTN), (b) high systolic BP (i.e., ≥130 mmHg) (SBP-HTN), (c) high diastolic BP (i.e., ≥80 mmHg) (DBP-HTN), and (d) high systolic and diastolic BP (BP-HTN). These groups were created to allow an exploratory analysis of HTN-related measures and cognitive, mood, and behavior parameters, since hypertension might be overdiagnosed due to the white coat effect, BP assessment tool, BP variability, physicians’ conduct and compliance to guidelines, and participants’ mood (19–22). In addition, most people with hypertension have problems with adherence to the treatment (23). Then, to provide a current view of the participants’ status and to reduce the risk of report bias, we categorized participants into “normal high” BP groups according to the guidelines of the European Society of Cardiology (24).

Drugs were coded according to the Anatomical Therapeutic and Chemical codes, a classification system that assigns a unique code to medicines according to the organ or system it works on and how it works. Drugs included Benazepril, Captopril, Enalapril, Fosinopril, Lisinopril, Moexipril, Perindopril, Quinapril, Ramipril, and Trandolapril. Analytical variables were created for the use of ACEIs and other antihypertensive drugs.

### 2.4 Cognitive, mood, and behavioral aspects

Cognitive, mood, and behavioral aspects were assessed according to items of sections B and E of the Minimum Data Set Home Care (MDS-HC) instrument (25). Section B involves six questions distributed in three cognitive domains (i.e., memory recall ability, decision-making, and indicators of delirium) regarding participants’ perceptions of their cognitive function in the last weeks. Possible answers are binary (i.e., yes or no), except for item 2: *How well client made decisions about organizing the day (*e.g.*, when to get up or have meals, which clothes to wear or activities to do)?*, which includes five possible answers. A binary variable was created by diving those who reported being independent (i.e., 0) and dependents (i.e., 1). Section E contains 16 questions distributed in four complexes, which include indicators of depression, anxiety, and sad mood, mood decline, behavioral symptoms, and changes in behavioral symptoms. Except for item 2, which involves a binary answer, all other items include 3 potential responses: (a) not exhibited/not occurred, (b) exhibited 1–2 of last 3 days/occurred, easily altered, and (c) exhibited on each of last 3 days/occurred, not easily altered. Binary variables were created by separating answers “a” (i.e., 0) and answers “b” and “c” (i.e.,1). Participants’ self-reports were obtained during interviews conducted by healthcare professionals.

### 2.5 Inflammatory markers

Blood samples were obtained from approximately 97% of participants. Blood was drawn after overnight fasting by a trained phlebotomist according to a standardized protocol. Blood samples were immediately centrifuged at 4°C and stored at −80°C until analysis. C-reactive protein (CRP), interleukin-6 levels (IL-6), and tumor necrosis factor-α (TNFα) were measured in plasma by enzyme-linked immunosorbent assays (ELISA; High Sensitivity Quantikine KitR&D Systems, Minneapolis, MN). All assays were run in duplicate, and average values were used for the analyses.

### 2.6 Statistical analysis

Continuous variables are expressed as mean ± standard deviation (SD), while categorical variables are reported as absolute numbers and percentages. Comparisons between groups with and without hypertension (e.g., CLI-HTN and NON-CLI-HTN) were performed using independent t-tests. Chi-square (Χ^2^) tests and binary regression were used to explore the relationship between BP and cognitive, mood, and behavioral parameters. Χ^2^ tests were performed within each group (e.g., SBP-HTN) comparing the prevalence of the variables between hypertensive and normotensive participants. Regressions were performed using BP-related parameters as independent variables and each binary answer of the MDS-HC, as the dependent variable. Comparisons were performed between condition (e.g., CLI-HTN) and non-condition (e.g., NON-CLI-HTN), with the last used as the reference group. No comparisons were performed among the different classifications (e.g., CLI-HTN vs. SBP-HTN). The final models were adjusted for age, sex, BMI, physical activity levels, multimorbidity, smoking status, unintentional loss of weight, schooling years, antipsychotic and antidepressant drugs, and ACEI use. For all tests, the level of significance was set at 5% (*p* < 0.05). All *p* values were determined by two-tailed tests. Analyses were performed using the SPSS software (version 23.0, SPSS Inc., Chicago, IL, United States).

## 3 Results

The main characteristics of participants of the present study are shown in Table 1 for CLI-HTN, and in (Supplementary Table S1), for groups classified according to BP levels. Participants categorized as hypertensive according to BP values were younger, lighter, and more physically active than those normotensives. Hypertensive groups had higher systolic BP, whereas higher diastolic BP was observed in SBP-HTN, DBP-HTN, and BP-HTN groups. In the BP-HTN group, normotensive participants took more antipsychotic drugs than those with hypertension. As expected, a high prevalence of participants on ACEI was observed in the hypertensive subgroup of the CLI-HTN group.

**Table 1:** Main characteristics of study participants (*n* = 364).

Variables	Hypertensive (*n* = 185)	Normotensive (*n* = 179)	Total (*n* = 364)
Age (years)	85.5 ± 4.7	86.1 ± 4.9	85.8 ± 4.8
Women (*n*, %)	128 (69.2)	116 (64.8)	244 (67.0)
BMI (kg/m^2^)	25.7 ± 4.5	25.4 ± 4.5	25.6 ± 4.5
Weight (kg)	63.0 ± 12.4	62.3 ± 13.2	62.7 ± 12.8
Height (m)	1.56 ± 0.09	1.56 ± 0.09	1.56 ± 0.08
Current smoking (*n*, %)	40 (21.6)	44 (24.6)	84 (23.0)
Loss of weight (*n*, %)	36 (19.5)	33 (18.4)	69 (19.0)
Physically active (*n*, %)	33 (17.8)	32 (17.9)	65 (17.8)
SBP (mmHg)	148.4 ± 26.0	141.8 ± 23.1^*^	145.4 ± 24.8
DBP (mmHg)	81.6 ± 14.5	80.0 ± 11.6	81.0 ± 13.1
Multimorbidity, yes	2 (1.1)	9 (4.9)^*^	11 (3.0)
Antipsychotic drugs, yes	8 (2.2)	14 (3.8)	22 (6.0)
Antidepressant drugs, yes	4 (1.1)	4 (1.1)	8 (2.1)
Angiotensin-converting-enzyme inhibitors, yes	103 (55.7)	0 (0.0)^*^	103 (28.2)
Schooling	4 (1.1)	6 (1.6)	10 (2.7)
No schooling	1 (0.3)	1 (0.3)	2 (0.5)
8th grade/less	168 (46.2)	163 (44.8)	331 (90.9)
9–11 grades	6 (1.6)	3 (0.8)	9 (2.4)
High school	4 (1.1)	3 (0.8)	7 (1.9)
Technical or trade school	2 (0.5)	2 (0.5)	4 (1.0)
Some college	0 (0.0)	1 (0.3)	1 (0.2)

Data are shown as mean ± SD and *n* (%). ^*^p < 0.05 vs Hypertensive.

Table 2 shows the results for the associations between hypertension-related parameters and cognitive function, mood, and behavioral aspects. Hypertensive participants reported fewer problems with short-term and procedural memory and worsening in decision-making in comparison to normotensive participants, regardless of the categorization method, except for procedural memory in the CLI-HTN subgroup. In the SBP-HTN subgroup, hypertensive participants had a lower frequency of changes in mental function. Regarding mood and behavioral aspects, hypertensive participants of the BP-HTN subgroup reported experiencing fewer feelings of sadness, unrealistic fears, repetitive health and anxious complaints, displayed more facial expressions of sadness, had more episodes of recurrent crying, withdrawal from activities of interest, and reduced social interaction. Persistent anger was mostly observed in hypertensive participants categorized according to systolic and diastolic BP. In the CLI-HTN and DBP-HTN subgroups, hypertensive participants reported having more recurrent crying. Furthermore, hypertensive participants in the CLI-HTN had reduced social interaction. No other significant associations were observed.

**Table 2:** Non-adjusted associations between hypertension-related parameters and cognitive function, mood, and behavioral aspects.

	Hypertensive	SBP-HTN	DBP-HTN	BP-HTN
Cognition				
Problems with short-term memory, yes	46 (12.6)^*^	47 (12.9)^*^	67 (18.4)^*^	3 (0.8)^*^
Problems with procedural memory, yes	29 (8.0)	36 (9.9)^*^	35 (9.6)^*^	1 (0.3)^*^
Worsening in decision making, yes	43 (11.8)^*^	52 (14.3)^*^	54 (14.8)^*^	0 (0.0)^*^
Changes in mental function, yes	2 (0.5)	1 (0.3)^*^	2 (0.5)	0 (0.0)
Agitation and disorientation, yes	4 (1.1)	3 (0.8)	4 (1.1)	0 (0.0)
Mood and behavior				
Feelings of sadness or being depressed, yes	60 (16.5)	72 (19.8)	75 (20.6)	7 (1.9)^*^
Persistent anger, yes	5 (1.4)	13 (3.6)^*^	13 (3.6)^*^	0 (0.0)
Unrealistic fears, yes	19 (5.2)	27 (7.4)	24 (6.6)	1 (0.3)^*^
Repetitive health complaints, yes	39 (10.7)	51 (14.0)	48 (13.2)	2 (0.5)^*^
Repetitive anxious complaints, yes	28 (7.7)	37 (10.2)	34 (9.3)	2 (0.5)^*^
Facial expressions of sadness, worry, and pain, yes	38 (10.4)	48 (13.2)	48 (13.2)	2 (0.5)^*^
Recurrent crying, yes	23 (6.3)^*^	27 (7.4)	28 (7.7)^*^	2 (0.5)^*^
Withdrawal from activities of interest, yes	41 (11.3)	48 (13.2)	45 (12.4)	2 (0.5)^*^
Reduced social interaction, yes	41 (11.3)^*^	45 (12.4)	41 (11.3)	1 (0.3)^*^
Mood decline, yes	0 (0.0)	1 (0.3)	0 (0.0)	0 (0.0)
Wandering, yes	0 (0.0)	1 (0.3)	1 (0.3)	1 (0.3)
Symptoms of verbally abuse behavior, yes	0 (0.0)	1 (0.3)	1 (0.3)	0 (0.0)
Symptoms of physically abuse behavior, yes	0 (0.0)	1 (0.3)	1 (0.3)	0 (0.0)
Inappropriate/Disruptive social behavior, yes	0 (0.0)	2 (0.5)	4 (1.1)	2 (0.5)
Resists care, yes	0 (0.0)	2 (0.5)	2 (0.5)	0 (0.0)
Changes in behavior symptoms, yes	1 (0.3)	0 (0.0)	1 (0.3)	0 (0.0)

Data are shown as *n* (%). ^*^p < 0.05 for the chi-square test within the group. The term — yes — refers to the presence of the condition. Comparisons were performed between condition (e.g., CLI-HTN) versus non-condition (e.g., NON-CLI-HTN), with the last used as the reference group (omitted).

Results of the binary regression for the associations between hypertension-related parameters and cognitive function, mood, and behavioral aspects adjusted for covariates are shown in Table 3. We observed that hypertensive participants in the CLI-HTN and BP-HTN groups had a lower likelihood of having problems with short-term memory. Hypertensive participants of the BP-HTN subgroup had also lesser reductions in social interactions, whereas those in the CLI-HTN reported more reduced social interaction. Furthermore, high systolic BP (continuous) was significantly associated with fewer reports of worsening in decision-making. Hypertensive participants who had high systolic and diastolic BP values (BP-HTN) reported fewer feelings of sadness and depressive symptoms, unrealistic fears, and anxious complaints, as well as showed fewer facial expressions associated with feelings of sadness, pain, and worry, and had a lesser withdrawal from activities of interest. In contrast, hypertensive participants categorized according to systolic BP reported more episodes of recurrent crying.

**Table 3:** Associations between hypertension-related parameters and cognitive aspects.

	Adjusted OR (95% CI)*	*p*-value
Problems with short-term memory		
**CLI-HTN**	**0.52 (0.29, 0.93)**	**0.003**
SBP-HTN	0.87 (0.46, 1.65)	0.679
DBP-HTN	1.01 (0.53, 1.92)	1.017
**BP-HTN**	**0.09 (0.01, 0.44)**	**0.003**
Problems with procedural memory		
SBP-HTN	0.98 (0.42, 2.26)	0.964
DBP-HTN	1.21 (0.51, 2.83)	0.660
BP-HTN	0.10 (0.01, 1.59)	0.104
Worsening in decision making		
BP-HTN	0.000	0.995
DBP-HTN	0.94 (0.47, 1.86)	0.862
**SBP**	**0.98 (0.97, 0.99)**	**0.034**
Changes in Mental Function		
SBP-HTN	6.63 (0.29, 149.7)	0.234
Feelings of Sadness and Depressive Symptoms		
**BP-HTN**	**0.24 (0.10, 0.62)**	**0.003**
Persistent Anger		
SBP-HTN	0.000	0.999
DBP-HTN	0.000	0.999
Unrealistic Fears		
**BP-HTN**	**0.11 (0.01, 0.90)**	**0.040**
Anxious Complaints		
**BP-HTN**	**0.19 (0.04, 0.92)**	**0.040**
Sad, Pained, Worried Facial Expressions		
**BP-HTN**	**0.09 (0.02, 0.45)**	**0.003**
Reccurent crying, tearfulness		
CLI-HTN	2.30 (0.98, 5.40)	0.054
**SBP-HTN**	**5.43 (1.43, 20.6)**	**0.013**
Withdrawal from Activities of Interest		
**BP-HTN**	**0.09 (0.02, 0.44)**	**0.003**
Reduced Social Interaction		
**CLI-HTN**	**1.94 (1.02, 3.67)**	**0.042**
**BP-HTN**	**0.05 (0.01, 0.41)**	**0.005**

^*^Models adjusted for age, sex, body mass index, multimorbidity, unintentional loss of weight, smoking habits, schooling years, physical activity and antipsychotic and antidepressant drugs. Bold indicates significance according to *p*-values and confidence interval. Comparisons were performed between condition (e.g., CLI-HTN) versus non-condition (e.g., NON-CLI-HTN), with the last used as the reference group.

Table 4 shows binary regression results for the association between hypertension-related parameters and cognitive function, mood, and behavioral aspects according to ACEI use. After adjusting the analysis for ACEI use, only the associations between hypertension and lesser problems with short-term memory in CLI-HTN and BP-HTN remained significant.

**Table 4:** Associations between hypertension-related parameters and cognitive aspects according to ACEI use.

	Adjusted OR (95% CI)	*p*-value
Problems with short-term memory		
**CLI-HTN**	**0.47 (0.25, 0.88)**	**0.019**
**BP-HTN**	**0.08 (0.01, 0.43)**	**0.003**
Worsening in decision making		
SBP	1.056 (0.979, 1.140)	0.157
Feelings of Sadness and Depressive Symptoms		
BP-HTN	0	0.999
Unrealistic Fears		
BP-HTN	0	0.999
Anxious Complaints		
BP-HTN	1.45 (0.36, 5.81)	0.597
Sad, Pained, Worried Facial Expressions		
BP-HTN	1.23 (0.37, 4.09)	0.736
Reccurent crying, tearfulness		
SBP-HTN	3.54 (0.77, 16.1)	0.103
Withdrawal from Activities of Interest		
BP-HTN	0.75 (0.23, 2.40)	0.752
Reduced Social Interaction		
CLI-HTN	1. 80 (0.88, 3.65)	0.104
BP-HTN	1.03 (0.30, 3.47)	0.953

^*^Models adjusted for age, sex, body mass index, multimorbidity, unintentional loss of weight, smoking habits, physical activity, schooling years, and antipsychotic and antidepressant drugs. Bold indicates significance according to *p*-values and confidence interval. Comparisons were performed between condition (e.g., CLI-HTN) versus non-condition (e.g., NON-CLI-HTN), with the last used as the reference group.

**Figure 1:**
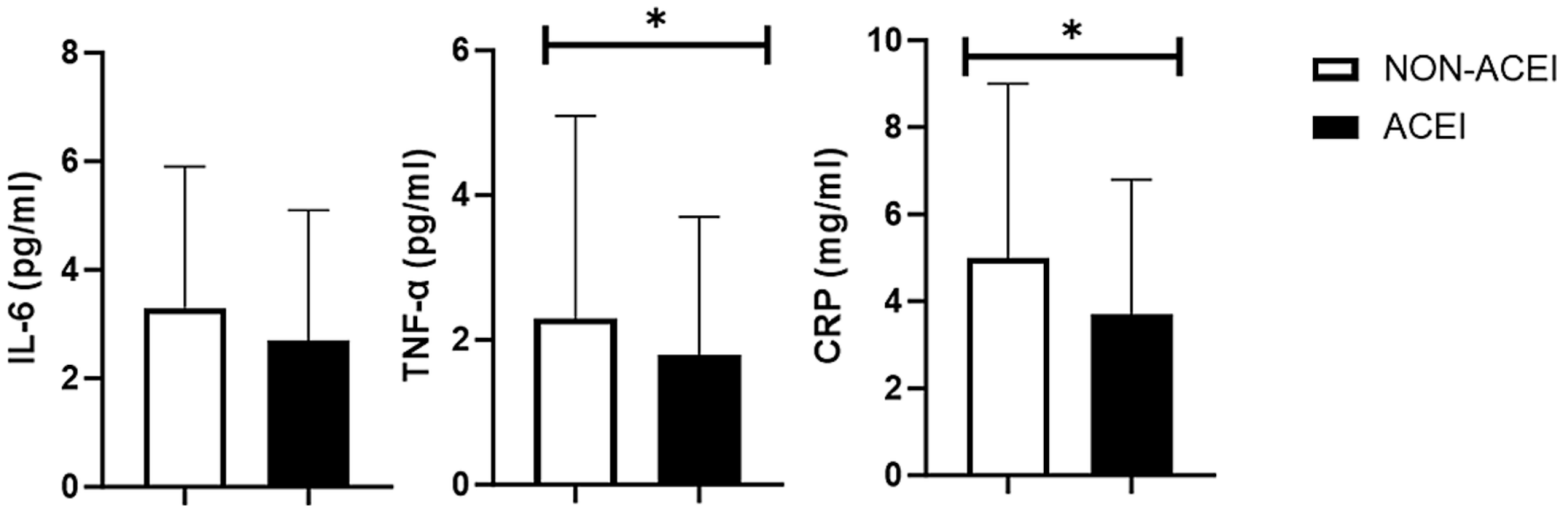
Blood concentration of interleukin 6 (IL-6), tumor necrosis factor alpha (TNF-α), and C-reactive protein according to the use of ACE-inhibitors. * *p* < 0.05.

Blood concentrations of inflammatory markers according to HTN are shown in Figure 1. Hypertensive participants in the BP-HTN group had lower IL-6 and CRP levels in comparison to normotensive peers. No other significant differences were observed.

## 4 Discussion

Findings of the present study indicate that HTN-related parameters are associated with many cognitive, mood, and behavioral aspects in very old adults. However, only associations with problems in short-term memory remained significant after adjusting the analysis for ACEI use. When possible candidate biomarkers to explain this scenario were examined, hypertensive participants, categorized according to BP values, had lower blood concentrations of IL-6 and CRP.

Our findings are partially supported by other studies that reported inverse associations between BP levels and cognitive function in very old adults. Nakamura et al. (6) found that high BP was a predictor of better global cognitive function 3 years later in a cohort of very old Japanese adults. Similar results were reported by Corrada et al. (7) and Chen et al. (26) after examining North American and Japanese older adults aged 90 years and older, respectively. Ruitenberg et al. (8) observed that lower systolic and diastolic BP values at baseline were associated with a higher risk of dementia in Dutch older adults. Another important finding was that reductions in BP during the follow-up were associated with the occurrence of dementia (8). Verghese et al. (9) followed up older adults over a median of 6.7 years and observed that each 10-mmHg decrement in BP was associated with a significant increase in the risk of dementia. Furthermore, individuals with mild to moderate systolic hypertension had a reduced risk of developing AD compared white those with normal BP (9). These findings were expanded by Li et al. (27), who showed that the association between BP and cognitive deterioration varies with age, given that high BP was a significant predictor of dementia in young older adults (approximately 60–70 years), whereas a negative association among these factors were found in those very old (≥85 years).

A possible explanation for these findings is that high BP might be a compensatory mechanism to maintain adequate cerebral perfusion in the face of vascular detriments caused by aging (6–9). Indeed, the old vessel is commonly characterized by losses in structural integrity and function, which involves significant impairment of endothelium dilation, resulting in reduced blood flow to major tissues, including the brain (28–30). In preclinical models, low cerebral blood flow (CBF) destroys the blood–brain barrier ultrastructure, causes microglial activation, increases the expression of proinflammatory markers, provokes overexpression of β-amyloid precursor protein, and reduces spatial learning and memory abilities (31–33).

Low CBF, regardless of the brain region affected, is associated with a higher risk of cognitive decline in hypertensive people and in mixed samples (34, 35). Furthermore, hypoperfusion of the medial temporal and posterior cingulate cortexes is found in individuals with memory impairments (35). In contrast, a high CBF is associated with greater hippocampal and amygdalar volumes and lower dementia risk (35). This scenario led experts in the field to propose the vascular hypothesis of AD, an alternative or complementary (or even substitutive) view to the classical neurodegenerative disorder model, in which it is stated that chronic insufficient brain perfusion underlies the metabolic and physiopathological aspects of AD (36).

Another important result of the present study was that hypertension-related parameters were no longer significantly associated with many cognitive, mood, and behavioral aspects when analyses were further adjusted for ACEI use. ANGII might contribute to cognitive decline and mood alteration by affecting cerebral structure and function. ANGII reduces CBF in response to vasodilator agents (e.g., bradykinin), disrupts the integrity of the blood–brain barrier, causes loss of pericytes and capillaries, and increases the cleavage of the amyloid precursor protein and amyloid deposition (12–14). This scenario induces microglia activation (13) and increases the expression of inflammatory and oxidative stress markers in brain cells (12, 13, 37). Although these changes have been noted in both young and old animals, aging exacerbates the effects of ANGII-induced hypertension on cerebral dysfunction (13).

On the other hand, ACEIs increase CBF, maintain the integrity of white and gray matter, reduce the presence and expression of inflammatory and oxidative markers in the brain, and increase neurotrophins levels (15–17). Furthermore, a growing number of investigations have reported that ACEI treatment might improve traits of cognitive dysfunction and psychiatric diseases in animal models, including immobility time, social deficits, and spatial learning (17, 38). In humans, those on ACEI treatment have a lower risk of cognitive decline, MCI, and AD (3–5). These results have been confirmed by systematic reviews and meta-analyses (33, 34). Regarding psychiatric aspects, individuals taking ACEI were at a lower risk of admission due to mood disorders in comparison to their peers taking other antihypertensive medications (35).

Taken together, these findings suggest that hypertensive participants on ACEI treatment might have fewer complaints about cognitive, mood, and behavioral problems in comparison to their counterparts taking other antihypertensive drugs. Such results could be mediated by the effects of ACEI on inflammatory markers (12, 13, 37) and explain the lower blood concentrations of IL-6 and CRP found in hypertensive older adults of the BP-HTN group. However, when we compared hypertensive ACEI users and non-users, significant differences in the *agitation and disorientation* item were observed (Supplementary Table S2). These findings suggest that other parameters might have influenced inflammation and better cognitive function in the hypertensive group (35). Nevertheless, the small sample size (<200 participants) and the lack of adjustments for covariates indicate that results should be carefully extrapolated and that more studies examining this scenario are still needed.

The present study is not free of limitations. First, cognitive, mood, and behavioral aspects were assessed using self-reported instruments, and no specific screening and diagnosis instruments were used. Second, results were not adjusted according to the onset of HTN (7). Third, participants were not screened according to the presence of the apolipoprotein E4 allele, which might influence our results (36). Fourth, blood concentrations of other possible mediators (e.g., reactive oxygen species) were not measured. Fifth, other aspects of ACEI treatment, such as dosage, duration, and adherence were not recorded (37, 38). Sixth, it is possible that the effects of ACEI on cognitive function are mediated by their capacity to cross the blood–brain barrier. This information was only available for some participants of the present study which prevented us from conducting further analysis. Seventh, the cross-sectional design of the study does not allow any inference to be drawn on the time course of changes in the variables considered or on cause–effect relationships. Eight, data regarding adherence to the antihypertensive pharmacological therapy was not recorded. Ninth, no screening instruments (e.g., mini-mental state examination) were used to examine participants’ cognitive status. Tenth, the lack of a detailed description of the antihypertensive treatment (e.g., use of diuretics) prevented us from conducting deeper analyses. Eleventh, the use of an oscillometric device might have produced different results (39). Finally, we examined a cohort of very old adults who lived in a mountain region, and extrapolations to other groups should be made with caution.

## Data availability statement

The raw data supporting the conclusions of this article will be made available by the authors, without undue reservation.

## Ethics statement

The studies involving humans were approved by Ethic Committee of the Università Cattolica del Sacro Cuore (Rome, Italy). The studies were conducted in accordance with the local legislation and institutional requirements. The participants provided their written informed consent to participate in this study.

## Author contributions

HJC-J: Conceptualization, Data curation, Formal analysis, Investigation, Methodology, Writing – original draft, Writing – review & editing. RC: Conceptualization, Data curation, Formal analysis, Investigation, Writing – original draft. MT: Conceptualization, Investigation, Writing – original draft. AR: Investigation, Writing – review & editing. FL: Conceptualization, Formal analysis, Funding acquisition, Investigation, Methodology, Resources, Supervision, Writing – original draft, Writing – review & editing. AP: Conceptualization, Formal analysis, Investigation, Methodology, Writing – original draft, Writing – review & editing. EM: Conceptualization, Data curation, Formal analysis, Investigation, Methodology, Resources, Supervision, Writing – original draft, Writing – review & editing.
