# Supplementary material for: Associations between hypertension and cognitive, mood, and behavioral parameters in very old adults: results from the IlSIRENTE study
Source: Front Public Health. 2024 Mar 12;11:1268983. doi: 10.3389/fpubh.2023.1268983 (PMC10964923; doi:10.3389/fpubh.2023.1268983)
Supplement: Supplementary file 1 [file Table_1.docx]

| Table SM1. Main characteristics of study participants (n=364) | | | | | | |
| --- | --- | --- | --- | --- | --- | --- |
|  | **SBP-HTN (n=251)** | **Non- SBP-HTN (n=113)** | **DBP-HTN (n= 245)** | **Non- DBP-HTN (n= 119)** | **BP-HTN (n= 212)** | **Non-BP-HTN (n= 80)** |
| Age (years) | 85.4 ± 4.4 | 86.9 ± 5.6a | 85.0 ± 4.2 | 87.4 ± 5.5b | 85.0 ± 4.1 | 87.6 ± 5.8c |
| Female (n, %) | 166 (66.1) | 78 (69.0) | 165 (67.3) | 79 (66.4) | 144 (67.9) | 57 (71.3) |
| BMI (kg/m^2^) | 25.9 ± 4.2 | 24.9 ± 5.0a | 26.3 ± 4.5 | 24.0 ± 4.2b | 26.1 ± 4.2 | 23.8 ± 4.3c |
| Weight (kg) | 63.3 ± 12.1 | 60.6 ± 14.0 | 64.4 ± 12.7 | 58.9 ± 12.2b | 63.8 ± 12.1 | 57.1 ± 11.7c |
| Height (m) | 1.56 ± 0.09 | 1.55 ± 0.09 | 1.56 ± 0.09 | 1.56 ± 0.09 | 1.56 ± 0.09 | 1.54 ± 0.09 |
| Current smoking (n, %) | 59 (23.5) | 25 (22.1) | 56 (22.9) | 28 (23.5) | 49 (23.1) | 18 (22.5) |
| Loss of weight (n, %) | 44 (17.5) | 25 (22.1) | 42 (17.1) | 27 (22.7) | 37 (17.5) | 20 (25.0) |
| Physically active (n, %) | 52 (20.7) | 13 (11.5) | 49 (20.0) | 16 (13.4)b | 45 (21.2) | 9 (11.3)c |
| SBP (mmHg) | 157.2 ± 19.0 | 117.9 ± 10.9a | 154.1 ± 22.2 | 126.3 ± 18.9b | 159.0 ± 19.7 | 115.8 ± 11.4c |
| DBP (mmHg) | 85.2 ± 12.5 | 71.1 ± 8.6a | 87.1 ± 11.0 | 68.0 ± 6.1b | 88.0 ± 11.5 | 66.9 ± 6.5c |
| Multimorbidity, yes | 6 (2.4) | 5 (4.4) | 6 (2.4) | 5 (4.2) | 5 (2.4) | 4 (5.0) |
| Antipsychotic drugs, yes | 12 (3.3) | 10 (2.7) | 12 (3.3) | 10 (2.7) | 0 (0.0) | 22 (6.0) |
| Antidepressant drugs, yes | 26 (7.1) | 30 (8.2) | 34 (9.3) | 22 (6.0) | 5 (1.4) | 51 (14.0) |
| Angiotensin-converting-enzyme inhibitors, yes | 103 | 34 | 91 | 43 | 81 | 24 |
| Schooling | 9 (2.5) | 1 (0.3) | 8 (2.2) | 2 (0.5) | 9 (2.5) | 1 (0.3) |
| No schooling | 2 (0.5) | 0 (0.0) | 2 (0.5) | 0 (0.0) | 2 (0.5) | 0 (0.0) |
| 8th grade/less | 224 (61.5) | 107 (29.4) | 215 (59.1) | 116 (31.9) | 269 (73.9) | 62 (17.0) |
| 9-11 grades | 6 (1,6) | 3 (0.8) | 8 (2.2) | 1 (0.3) | 5 (1.4) | 4 (1.1) |
| High school | 5 (1.4) | 2 (0.5) | 7 (1.9) | 0 (0.0) | 6 (1.6) | 1 (0.3) |
| Technical or trade school | 4 (1.1) | 0 (0.0) | 4 (1.1) | 0 (0.0) | 4 (1.1) | 0 (0.0) |
| Some college | 1 (0.3) | 0 (0.0) | 1 (0.3) | 0 (0.0) | 1 (0.3) | 0 (0.0) |

Data are shown as mean ± SD and n (%). aP<0.05 vs SBP-HTN, bP<0.05 vs DBP-HTN, and cP<0.05 vs BP-HTN. SBP= Systolic blood pressure, DBP= Diastolic blood pressure.
