# Supplementary material for: Associations between hypertension and cognitive, mood, and behavioral parameters in very old adults: results from the IlSIRENTE study
Source: Front Public Health. 2024 Mar 12;11:1268983. doi: 10.3389/fpubh.2023.1268983 (PMC10964923; doi:10.3389/fpubh.2023.1268983)
Supplement: Supplementary file 2 [file Table_2.docx]

| **Table SM2.** Main characteristics of hypertensive participants according to ACEI use (n=185) | | |
| --- | --- | --- |
| Variables |  | |
|  | **ACEI-HTN (n= 103)** | **NON- ACEI-HTN (n= 82)** |
| Age (years) | 85.3 ± 4.4 | 85.9 ± 5.1 |
| Female (n, %) | 37 (20.0) | 20 (10.8) |
| BMI (kg/m^2^) | 26.9 ± 4.7 | 25.5 ± 4.3 |
| Weight (kg) | 64.3 ± 4.7 | 61.3 ± 11.5 |
| Height (m) | 1.57 ± 0.0 | 1.54 ± 0.0 |
| Current smoking (n, %) | 22 (11.8) | 18 (9.7) |
| Loss of weight (n, %) | 22 (11.8) | 14 (7.5) |
| Physically active (n, %) | 19 (10.2) | 13 (7.0) |
| SBP (mmHg) | 150.1 ± 26.8 | 146.2 ± 24.4 |
| DBP (mmHg) | 82.2 ± 16.5 | 80.8 ± 11.7 |
| Multimorbidity, yes | 5 (2.7) | 4 (2.1) |
| Antipsychotic drugs, yes | 2 (1.1) | 9 (4.9) |
| Antidepressant drugs, yes | 8 (4.3) | 14 (7.6) |
| Schooling |  |  |
| No schooling | 1 (0.5) | 3 (1.6) |
| 8-11 grade/less | 1 (0.5) | 0 (0.0) |
| 8-11 grades/less | 79 (42.2) | 90 (48.6) |
| High school, Techinical | 1 (0.5) | 5 (2.7) |
| Technical or trade school | 1 (0.5) | 3 (1.6) |
| Some college | 1 (0.5) | 2 (1.1) |
| IACE= Angiotensin-converting enzyme inhibitors; Data are shown as mean ± SD and n (%). | | |
